# Reducing Occurrence and Severity of Pneumonia Due to Pandemic H1N1 2009 by Early Oseltamivir Administration: A Retrospective Study in Mexico

**DOI:** 10.1371/journal.pone.0021838

**Published:** 2011-07-08

**Authors:** Anjarath Lorena Higuera Iglesias, Koichiro Kudo, Toshie Manabe, Alexander Enrique Corcho Berdugo, Ariel Corrales Baeza, Leticia Alfaro Ramos, René Guevara Gutiérrez, María Eugenia Manjarrez Zavala, Jin Takasaki, Shinyu Izumi, Edgar Bautista, José Rogelio Perez Padilla

**Affiliations:** 1 National Institute for Respiratory Disease, Mexico City, Mexico; 2 National Center for Global Health and Medicine, Tokyo, Japan; University of Liverpool, United Kingdom

## Abstract

**Background:**

Anti-viral treatment has been used to treat severe or progressive illness due to pandemic H1N1 2009. A main cause of severe illness in pandemic H1N1 2009 is viral pneumonia; however, it is unclear how effective antiviral treatment is against pneumonia when administered >48 hours after symptom onset. Therefore, we aimed to determine how time from symptom onset to antiviral administration affected the effectiveness of antiviral treatment against pneumonia due to pandemic (H1N1) 2009.

**Methods/Principal Findings:**

A retrospective medical chart review of 442 patients was conducted in a hospital in Mexico. Subjects had tested positive for pandemic H1N1 2009 virus by real-time reverse-transcriptase-polymerase-chain-reaction and were administered oseltamivir. Median time from symptom onset to oseltamivir administration was 5.0 days (range, 0–43). 442 subjects, 71 (16.1%) had severe pneumonia which required mechanical ventilation, 191 (43.2%) had mild to moderate pneumonia, and 180 (40%) did not have pneumonia. Subjects were divided into four groups based on time to oseltamivir administration: ≤2, 3–7, 8–14, and >14 days. Severity of respiratory features was associated with time to treatment, and multivariate analysis indicated that time to oseltamivir administration was associated with severity of respiratory features. A proportional odds model indicated that 50% probability for occurrence of pneumonia of any severity and that of severe pneumonia in patients who would develop pneumonia reached at approximately 3.4 and 21 days, respectively, after symptom onset. Patients with a shorter time to oseltamivir administration were discharged earlier from the hospital.

**Conclusions:**

Earlier initiation of oseltamivir administration after symptom onset significantly reduced occurrence and severity of pneumonia and shortened hospitalization due to pandemic H1N1 2009. Even when administered >48 hours after symptom onset, oseltamivir showed considerable potential for reducing pneumonia. Application of these results would benefit patients affected by future influenza pandemics.

## Introduction

Pandemic H1N1 2009 emerged in Mexico in March 2009 [1] and rapidly spread throughout the world. The fatality rate and the frequency of severe cases varied among countries and regions even among different regions within the same country [2], [3]. Mexico reported a number of cases with severe clinical presentations and deaths, especially in the early period of the outbreak. This occurred at least in part because the novelty of the influenza strain was not recognized until April 23, 2009 [1], [4]. The World Health Organization [5] and the Center for Disease Control and Prevention [6] recommended early initiation of treatment with antiviral drugs in patients with pandemic H1N1 2009 virus infection with severe or progressive illness as well as in high-risk populations. The main cause of severe illness in pandemic H1N1 2009 was viral pneumonia [1], [3], [7], [8], [9] which is relatively rare in seasonal influenza. Oseltamivir has been used to treat influenza virus infection. The efficacy of oseltamivir treatment commencing >48 h after symptom onset in seasonal influenza has not been established [10]. There has been concern whether the time-interval from symptom onset to administration of oseltamivir affects clinical features on patients with pandemic H1N1 2009 virus infection. This study investigated how the post-onset window to oseltamivir administration affected the occurrence and severity of pneumonia and the duration of hospitalization in patients treated at the National Institute of Respiratory Diseases (INER) in Mexico City, Mexico during the pandemic period.

## Materials and Methods

### Study design

INER is a national tertiary care organization that includes a research center devoted to respiratory diseases. INER provides medical services primarily for economically deprived and uninsured populations, many of whom are from the Mexico City Metropolitan area. Medical records of patients with RT-PCR confirmed pandemic H1N1 2009 infections who were treated at the INER between April 1, 2009 and January 31, 2010 and were administered oseltamivir, the only available antiviral drug in the INER during the pandemic period, were retrospectively reviewed. All patients who were administered oseltamivir during the study period were included in this study. The regular dosage and duration of oseltamivir treatment was 150 mg/day for 5 days. However, the duration was extended when deemed necessary because of the patient's clinical conditions.

Clinical data, chest radiologic findings and laboratory findings were reviewed in terms of time from symptom onset to oseltamivir administration. Socioeconomic background of patients was classified into 6 levels based on their daily income. The study patients were divided into the following 4 groups based on the number of days from symptom onset to oseltamivir administration: Group 1 (≤2 days), Group 2 (3–7 days), Group 3 (8–14 days), and Group 4 (>14 days). The groups were compared in terms of clinical symptoms and findings, severity of the respiratory features and the duration of hospitalization. Severity of respiratory features was classified into three categories: severe pneumonia, mild to moderate pneumonia, or upper respiratory tract involvement without pneumonia. Pneumonia was confirmed on the basis of abnormal shadows on chest radiographs and was considered severe if it required mechanical ventilation, and mild to moderate if it did not. The third category consisted of upper respiratory tract involvement without pneumonia. How these aspects were influenced by the time to oseltamivir administration from symptom onset was subsequently examined.

The study was approved by the Institutional Review Boards of the INER, Mexico and National Center for Global Health and Medicine, Japan. Written informed consent was obtained from all hospitalized study patients or their relatives and verbal consent from all study outpatients was obtained in accordance with the Review Boards in the INER. Investigators kept the datasets in password-protected systems and presented data with the anonymity of study patients.

### Statistical analysis

Statistical analyses were performed by comparing Groups 1 through 4, and testing the linearity of incidence. Patient background data (qualitative and quantitative values) and clinical laboratory values were summarized, and the linearity of incidence of each symptom according to the number of days to oseltamivir administration was analyzed using Cochran-Armitage test. The severity of the respiratory features was analyzed using Jonckheere's test. Severe pneumonia, mild to moderate pneumonia, and upper respiratory tract involvement without pneumonia were handled as ordinal variables and were analyzed using proportional odds models with the time-interval from the symptom onset to the oseltamivir administration as the explanatory and continuous variable [11]. A graph of the probability of severe pneumonia and the probability of mild to severe (any severity) pneumonia, which were estimated by varying the number of days from the symptom onset from 0 to 50 days with the estimated regression coefficient, was depicted. The calculation formula was provided in the figure legend. The multivariate analysis using a proportional odds model was performed to identify profound factors that affected the severity of respiratory features. The explanatory variables were Groups 1 through 4, the socioeconomic background, gender, age and comorbidities.

The cumulative rate of hospital discharge for Groups 1 through 4 was calculated by Kaplan-Meier's method using mortality data as censored data and then comparatively analyzed using Tarone's test.

Data analyses were conducted using SAS version 9.2 (SAS Institute Inc., Cary, North Carolina). For all analyses, significance levels were two tailed, and a P value of <0.05 was considered significant.

## Results

### Characteristics of the study patients

Among patients suspected of having pandemic H1N1 2009 virus infection and admitted to the INER during the study period, 528 were confirmed to have pandemic H1N1 2009 virus infection by RT-PCR. There were no resistant viral strains involved in this study. Of these 528 patients, 442 received oseltamivir treatment during either ambulatory care (45.5%) or hospitalization (54.5%), these included 24 individuals who died. The 442 patients comprised the study subjects from whom clinical data were collected. Their characteristics are shown in Table 1. Data were obtained at the time of patients' presentation to the INER before oseltamivir administration. Median days from symptoms onset to oseltamivir administration was 5.0 (range, 0–43). Groups 1, 2, 3, and 4 comprised 92 (20.8%), 213 (48.2%), 101 (22.9%), and 36 (8.1%) patients, respectively.

**Table 1:** Characteristics of study patients.

Variable	Oseltamivir administration				Total	P value
Days from symptom onset until oseltamivir administration	Group 1≤2	Group 23–7	Group 38–14	Group 4>14	Median 5.0*(range, 0–43)	
No. of patients (% of all study patients)	n = 92 (20.8%)	n = 213 (48.2%)	n = 101 (22.9%)	n = 36 (8.1%)	n = 422 (100%)	
**Hospitalized/ambulatory, No. (% in each group)**						0.000^2^
Hospitalized	21 (22.8)	107 (50.2)	79 (78.2)	34 (94.4)	241 (54.5)	
Ambulatory	71 (77.2)	106 (49.8)	22 (21.8)	2 (5.6)	201 (45.5)	
**Deaths, No. (% in each group)**						0.004^4^
	1 (1.1)	10 (4.7)	9 (8.9)	4 (11.1)	24 (5.7)	
**Age (y), mean ± SD**	31.4±16	32.8±16.4	37.8±16.2	38.5±17.4	34±16.5	0.010^1^
Range	1.3–73.5	0.4–74.6	0.7–81.4	0.7–81.4	0–85	
**Age (y), No. (% in each group)**						
<1	0 (0.0)	5 (2.3)	2 (2.0)	1 (2.8)	8 (1.8)	
1 – <5	5 (5.4)	14 (6.6)	4 (4.0)	2 (5.6)	25 (5.7)	
5 – <10	4 (4.3)	5 (2.3)	2 (2.0)	0 (0.0)	11 (2.5)	
10 – <18	10 (10.9)	16 (7.5)	4 (4.0)	1 (2.8)	31 (7.0)	
18 – <50	60 (65.2)	141 (66.2)	67 (66.3)	24 (66.7)	292 (66.1)	
50 – <65	12 (13.0)	29 (13.6)	20 (19.8)	6 (16.7)	67 (15.2)	
≥65	1 (1.1)	3 (1.4)	2 (2.0)	2 (5.6)	8 (1.8)	
**Male sex, No. (% in each group)**	55 (59.8)	114 (53.5)	56 (55.4)	24 (66.7)	249 (56.3)	0.436^2^
**Socioeconomic background** † **, No. (% in each group)**						0.001^3^
0	5 (5.4)	12 (5.6)	19 (18.8)	6 (16.7)	42 (9.5)	
1	7 (7.6)	29 (13.6)	14 (13.9)	11 (30.6)	61 (13.8)	
2	75 (81.5)	146 (68.5)	61 (60.4)	14 (38.9)	296 (67.0)	
3	4 (4.3)	18 (8.5)	4 (4.0)	4 (11.1)	30 (6.8)	
4	0 (0.0)	4 (1.9)	3 (3.0)	1 (2.8)	8 (1.8)	
5	1 (1.1)	4 (1.9)	0 (0.0)	0 (0.0)	5 (1.1)	
**Influenza vaccination in 2008 and/or 2009, No. (% in each group)**	3 (3.3)	4 (1.9)	4 (4.0)	0 (0.0)	11 (2.5)	0.499^2^
**Comorbidities and others, No. (% in each group)**						
Obesity	7 (7.6)	28 (13.1)	15 (14.9)	4 (11.1)	54 (12.2)	0.442^2^
Diabetes	2 (2.2)	13 (6.1)	8 (7.9)	2 (5.6)	25 (5.7)	0.370^2^
Hypertension	5 (5.4)	17 (8.0)	11 (10.9)	5 (13.9)	38 (8.6)	0.357^2^
Chronic heart failure	0 (0.0)	2 (0.9)	1 (1.0)	0 (0.0)	3 (0.7)	0.745^2^
Asthma	10 (10.9)	22 (10.3)	5 (5.0)	5 (3.9)	42 (9.5)	0.307^2^
COPD‡	1 (1.1)	0 (0.0)	1 (1.0)	1 (2.8)	3 (0.7)	0.243^2^
Immunocompromised	0 (0.0)	2 (0.9)	3 (3.0)	1 (2.8)	6 (1.4)	0.256^2^
Steroid treatment	0 (0.0)	1 (0.5)	2 (2.0)	2 (5.6)	5 (1.1)	0.032^2^
Smoking	19 (20.7)	66 (31.0)	34 (33.7)	16 (44.4)	135 (30.5)	0.046^2^
Alcohol dependence	7 (7.6)	18 (8.5)	18 (17.8)	6 (16.7)	49 (11.1)	0.038^2^
Drug dependence	0 (0.0)	1 (0.5)	3 (3.0)	3 (8.3)	7 (1.6)	0.002^2^

1 One-way ANOVA,

2 Chi-square test,

3 Kruskal-Wallis test,

4 Cochran-Armitage test.

Grouping of patients was based on the number of days from symptom onset to oseltamivir administration: Group 1, ≤2 days; Group 2, 3–7 days; Group 3, 8–14 days; Group 4, >14 days.

*Median number of days from symptom onset to oseltamivir administration among all study patients.

†Socioeconomic background, based on patient's approximate daily income: 0 = <$5 US, 1 = $6–$10, 2 = $11–$15, 3 = $16–$25, 4 = $26–$40, 5 = >$40.

‡COPD: chronic obstructive pulmonary disease.

Median age of study patients (193 females and 249 males) was 36 years with a range of 4 months to 85 years. Of the 442 study patients, 75 (17%) were aged <18 years, and 292 (66.1%) were aged 18 to 50 years. A total of 399 (90.3%) earned less than $15 US per day (<level 2). Only 11 (2.5%) had a history of vaccination for seasonal influenza in 2008 and/or 2009. The number of hospitalized patients (P<0.001), mortality (P = 0.004), older age (P = 0.01), and lower socioeconomic level (P = 0.001) were associated with the Groups. The presence of comorbidities was not associated with the Groups; however, smoking (P<0.05), drug dependency (P = 0.002), and alcohol dependency (P<0.04) were associated.

Symptoms of the study patients were shown in Table 2. The main symptoms were cough (76.9%), arthralgia (52.0%), dyspnea (50.7%), myalgia (50.0%), purulent sputum (19.0%), cyanosis (17.0%), and sore throat(15.4%). A total of 59% of study patients had pneumonia with abnormal shadows on chest radiographs, and 16% required mechanical ventilation.

**Table 2:** Clinical features of the study patients on admission.

Variable	Oseltamivir administration				Total	P value*
Days from symptom onset until oseltamivir administration	Group 1≤2	Group 23–7	Group 38–14	Group 4>14		
No. of patients (% of all study patients)	n = 92 (20.8%)	n = 213 (48.2%)	n = 101 (22.9%)	n = 36 (8.1%)	n = 422 (100%)	
Abnormal respiratory sounds	17 (18.5)	78 (36.6)	48 (47.5)	13 (36.1)	156 (35.3)	<0.001
Hemoptysis	0 (0.0)	10 (4.7)	14 (13.9)	4 (11.1)	23 (6.3)	<0.001
Abnormal pulmonary shadows†	23 (25.0)	121 (56.8)	84 (83.2)	34 (94.4)	262 (59.3)	<0.001
Pneumothorax†	0 (0.0)	2 (0.9)	5 (5.0)	2 (5.6)	9 (2.0)	0.004
Pleurisy†	0 (0.0)	1 (0.5)	3 (3.0)	2 (5.6)	6 (1.4)	0.005
Chest pain	18 (19.6)	61 (28.6)	31 (30.7)	12 (33.3)	122 (27.6)	0.068
Dyspnea	27 (29.3)	100 (46.9)	70 (69.3)	27 (75.0)	224 (50.7)	<0.001
Cyanosis	2 (2.2)	33 (15.5)	29 (28.7)	11 (30.6)	75 (17.0)	<0.001
Intubation	2 (2.2)	31 (14.6)	26 (25.7)	12 (33.3)	71 (16.1)	<0.001
Vomiting	4 (4.3)	10 (4.7)	4 (4.0)	3 (8.3)	21 (4.8)	0.573
Diarrhea	3 (3.3)	11 (5.2)	6 (5.9)	4 (11.1)	24 (5.4)	0.105
Myalgia	45 (48.9)	112 (52.6)	48 (47.5)	16 (44.4)	221 (50.0)	0.540
Asthenia	7 (7.6)	41 (19.2)	20 (19.8)	10 (27.8)	78 (17.6)	0.006
Cough	62 (67.4)	168 (78.9)	79 (78.2)	31 (86.1)	340 (76.9)	0.027
Purulent sputum	13 (14.1)	36 (16.9)	25 (24.8)	10 (27.8)	84 (19.0)	0.018
Arthralgia	40 (43.5)	119 (55.9)	52 (51.5)	19 (52.8)	230 (52.0)	0.381
Chills	2 (2.2)	25 (11.7)	5 (5.0)	2 (5.6)	34 (7.7)	0.872
Nasal obstruction	16 (17.4)	21 (9.9)	4 (4.0)	1 (2.8)	42 (9.5)	0.001
Sore throat	21 (22.8)	34 (16.0)	11 (10.9)	2 (5.6)	68 (15.4)	0.004
Abdominal pain	0 (0.0)	3 (1.4)	2 (2.0)	0 (0.0)	5 (1.1)	0.568
Conjunctivitis	20 (21.7)	17 (8.0)	5 (5.0)	3 (8.3)	45 (10.2)	0.002

Grouping of patients was based on the number of days from symptom onset to oseltamivir administration: Group 1, ≤2 days; Group 2, 3–7 days; Group 3, 8–14 days; Group 4, >14 days.

*Cochran-Armitage test.

†Chest radiological findings.

Clinical symptoms and findings that increased significantly with time to oseltamivir administration (P<0.05) were included abnormal respiratory sounds; hemoptysis; abnormal pulmonary shadows, pneumothorax and pleurisy on chest radiographs; dyspnea; cyanosis; and need for intubation. On the other hand, incidence of nasal obstruction, sore throat, and conjunctivitis decreased as the time to oseltamivir administration increased.

Among the laboratory findings (Table 3), oxygen saturation (SpO_2_) measured by pulse oximetry decreased significantly as the time to oseltamivir administration increased. Although within the normal range, serum albumin decreased significantly, and serum sodium, platelets, and body temperature differed significantly among the Groups.

**Table 3:** Laboratory data and physical findings for study patients at presentation*.

Variable	Group 1≤2			Group 23–7			Group 38–14			Group 4>14			P value**
	N	Mean	SD	N	Mean	SD	N	Mean	SD	N	Mean	SD	
WBC (10^3^/µL)†	20	8.19	5.13	101	6.62	3.39	73	7.32	3.99	29	7.89	4.12	0.212
Neutrophil count (10^3^/µL)	17	5.45	4.53	70	4.67	3.07	45	5.51	4.37	24	4.70	2.73	0.596
Lymphocytes count (10^3^/µL)	17	0.81	0.51	70	1.12	0.76	45	1.25	0.84	24	1.27	1.11	0.264
RBC (10^6^/µL)‡	16	5.04	0.63	68	4.80	0.62	44	4.76	0.54	22	4.58	0.72	0.161
Hemoglobin (g/dL)	18	14.9	1.42	85	14.06	2.08	63	13.97	1.77	26	13.55	2.26	0.161
Platelets (10^3^/µL)	19	200.9	48.6	86	186.8	85.4	72	232.9	116.5	29	228.9	107.4	0.021
Albumin (d/dL)	14	4.29	0.46	59	3.86	0.50	41	3.65	0.48	18	3.50	0.72	<0.001
Sodium (mEq/L)	19	137.6	4.5	80	136.5	3.5	62	138.4	4.1	25	138.2	3.9	0.020
Body temperature (°C)	69	37.7	1.0	157	37.4	1.0	75	37.1	0.9	28	37.3	0.9	0.007
Respiratory rate	76	23.1	5.9	171	24.4	7.3	89	25.7	10.2	32	25.6	8.5	0.178
SpO_2_ §	80	92.3	4.3	182	90.0	7.1	82	85.8	9.6	32	86.1	12.1	<0.001

Grouping of patients was based on the number of days from symptom onset to oseltamivir administration: Group 1, ≤2 days; Group 2, 3–7 days; Group 3, 8–14 days; Group 4, >14 days.

† WBC: white blood cell count,

‡ RBC: red blood cell count,

§ SpO_2_: oxygen saturation measured by pulse oximetry in room air.

*Normal ranges are as follows: WBC, 4000–10 000; neutrophil count, 2200–8250; lymphocyte count, 1500–4000; RBC, 3.8–6.5; hemoglobin, 11.5–17.0; platelets, 150–400; albumin, 2.30–3.50; sodium, 138–150; respiratory rate, 12–20 per min in adults; SpO_2_, 92–98% at sea level.

**One-way ANOVA.

SD denotes standard deviation.

### Severity of respiratory features

Severe pneumonia was present in 71 (16.1%) patients. Mild to moderate pneumonia was present in 191 (43.2%), and upper respiratory tract involvement without pneumonia was present in 180 (40.7%) (Table 4).

**Table 4:** Severity of respiratory features in each group.

Variable	Oseltamivir administration				Total
Days from symptom onset until oseltamivir administration	Group 1≤2	Group 23–7	Group 38–14	Group 4>14	
No. of patients (% of total patients)	n = 92 (20.8%)	n = 213 (48.2%)	n = 101 (22.9%)	n = 36 (8.1%)	n = 442
Severe pneumonia* (% of group)	2 (2.2)	31 (14.6)	26 (25.7)	12 (33.3)	71 (16.1)
Moderate/mild pneumonia† (% of group)	21 (22.8)	90 (42.3)	58 (57.4)	22 (61.1)	191 (43.2)
Upper respiratory tract infection‡ (% of group)	69 (75)	92 (43.2)	17 (16.8)	2 (5.6)	180 (40.7)

(P<0.001, Jonckheere's test).

Grouping of patients was based on the number of days from symptom onset to oseltamivir administration: Group 1, ≤2 days; Group 2, 3–7 days; Group 3, 8–14 days; Group 4, >14 days.

*Severe pneumonia: abnormal shadows on chest radiographs and required mechanical ventilation.

† Moderate/mild pneumonia: abnormal shadows on chest radiographs but did not require mechanical ventilation.

‡ Upper respiratory tract infection: absence of pneumonia.

The proportions of patients with severe pneumonia or mild to moderate pneumonia increased from Groups 1 to 4 (Table 4). By the contrast, the proportions of patients with upper respiratory tract infection without pneumonia decreased from Groups 1 to 4. These results indicated that the severity of respiratory features reflected the time to oseltamivir administration (P<0.001, Jonckheere's test).

The results obtained by the multivariate analysis using a proportional odds model indicated that severity of respiratory features was also affected by socioeconomic level, gender, hypertension, obesity, asthma and smoking (Table 5). The proportional odds ratios for severities of respiratory features for Groups 2–4 were 5.17, 15.02, and 20.40 times higher, respectively, than that for Group 1. The grouping, which indicated days to oseltamivir administration, showed a high level of significance in terms of the severity of respiratory features.

**Table 5:** Multivariate analysis of the severity of respiratory features using a proportional odds model.

Parameter	Regression coefficient	Standard error	χ2	P value	Reference group	Odds ratio and 95% confidence interval
Intercept 1	4.42	1.62				
Intercept 2	7.39	1.66				
Group 2*3–7	1.64	0.30	29.49	<0.001	vs Group 1*	5.17 (2.86–9.37)
Group 3*8–14	2.70	0.35	59.59	<0.001	vs Group 1*	15.02 (7.55–29.89)
Group 4*>14	3.01	0.44	45.33	<0.001	vs Group 1*	20.40 (8.48–49.10)
Socioeconomic category†	−0.49	0.12	15.91	<0.001	-†	0.60 (0.47–0.77)
Gender (female)	0.80	0.21	14.03	<0.001	vs male	2.23 (1.46–3.40)
AGE 2‡	0.37	0.29	1.60	0.205	vs AGE 1‡	1.45 (0.81–2.59)
AGE 3‡	0.94	0.37	6.35	0.011	vs AGE 1‡	2.57 (1.23–5.38)
Diabetes§	−0.34	0.43	0.64	0.424	vs present	0.70 (0.30–1.65)
Hypertension§	−0.85	0.36	5.37	0.020	vs present	0.42 (0.20–0.87)
Obesity§	−1.76	0.32	29.52	<0.001	vs present	0.17 (0.09–0.32)
Asthma§	−1.04	0.34	8.99	0.002	vs present	0.35 (0.17–0.69)
Smoking§	−0.73	0.24	9.16	0.002	vs present	0.47 (0.29–0.77)
Alcoholism§	0.22	0.34	0.42	0.515	vs present	1.25 (0.63–2.44)

*Grouping of patients was based on the number of days from symptom onset to oseltamivir administration: Group 1, ≤2 days; Group 2, 3–7 days; Group 3, 8–14 days; Group 4, >14 days.

† The odds ratio of socioeconomic level was presented when the level was upped by one unit as a continuous variable.

‡ AGE 1 (age <18 y), AGE 2 (age 18–50 y), AGE 3 (≥50 y).

§ Comorbidities are compared between present and absent.

χ2: Chi-square test.

### Occurrence of pneumonia

**Figure 1:**
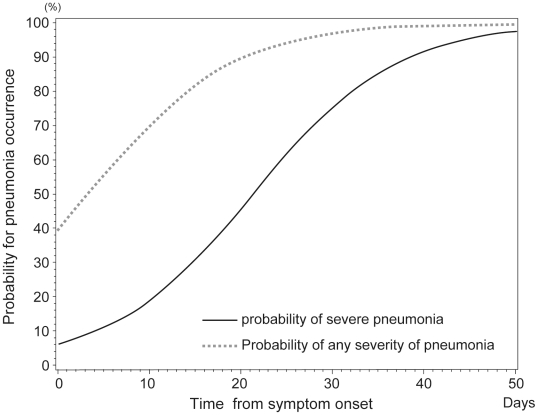
Model of the probability of occurrence of pneumonia. The probability of developing pneumonia is depicted using a proportional odds model. *p_1_*, the probability of severe pneumonia; *p_2_*, the probability of mild/moderate pneumonia; *x*, time until oseltamivir administration from symptom onset.
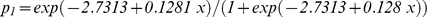


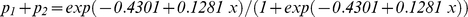



The number of patients who developed radiographic pneumonia was 262 (59.3%). The probability of pneumonia occurrence was estimated in relation to time from symptom onset using a proportional odds model (Figure 1). Figure 1 shows the time to occurrence of pneumonia. Both the probability of any severity of pneumonia and the probability of severe pneumonia increased gradually with the time from symptom onset to oseltamivir administration. The 50% probability for occurrence of any severity of pneumonia and that of severe pneumonia in patients who would develop pneumonia reached at approximately 3.4 and 21 days, respectively, after symptom onset.

The solid line indicates the probability of occurrence of severe pneumonia (*p_1_*). The dotted line indicates the probability of occurrence of any severity of pneumonia (mild to severe pneumonia, *p_1_+p_2_*).

### Duration of hospitalization

**Figure 2:**
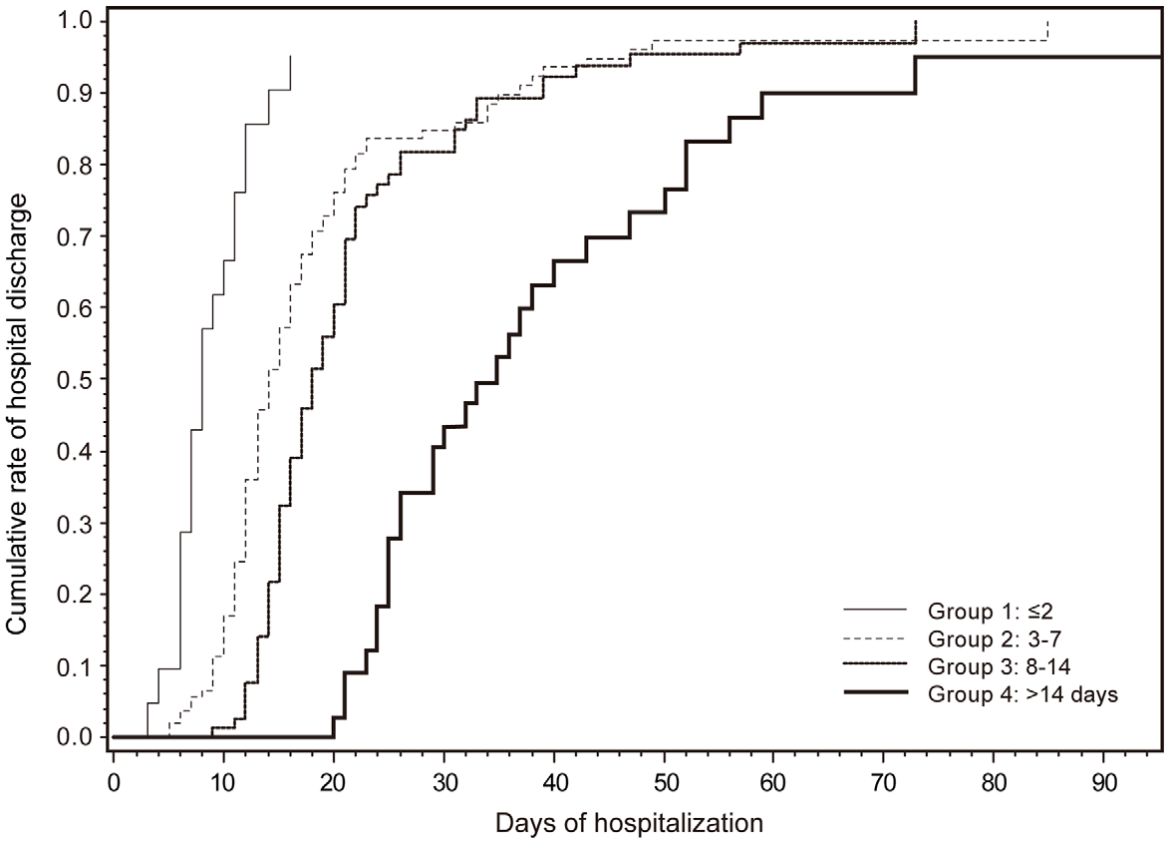
Cumulative rate of hospital discharge as a function of the time of oseltamivir administration. The cumulative rate of hospital discharge in each group using Kaplan-Meier's method was calculated using data on death as censored data. The groups of patients with earlier oseltamivir administration were discharged from the hospital significantly earlier (p<0.001, Tarone's test). Grouping of patients was based on days from symptom onset to oseltamivir administration: Group 1, ≤2 ; Group 2, 3–7; Group 3, 8–14; Group 4, >14days.

Of the 442 patients evaluated, 241 (54.5%) were hospitalized. The numbers of hospitalized patients in each group are shown in Table 1. The cumulative discharge rate in each group on a Kaplan-Meier's curve using death data as censored data is shown in Figure 2. Patients with a shorter time to oseltamivir administration were discharged earlier from the hospital (p<0.001, Tarone's test).

## Discussion

Severe pneumonia caused by pandemic H1N1 2009 has been frequently found [1], [3], [7], [8], [9], . Severity of pneumonia can be assumed to be a main indicator of overall disease severity. We hypothesized that the number of days to oseltamivir administration from symptom onset was one of the primary factors affecting the severity of pneumonia due to pandemic H1N1 2009.

Patients with pandemic H1N1 2009 infection in the INER had various time-intervals between the symptom onset and oseltamivir administration. Median days from symptom onset to oseltamivir administration was 5.0 (range 0–43) (Table 1), even though 92.5% of study patients was administered oseltamivir within one day after admission (median 0, range 0–12).

The study findings indicated that the following symptoms and clinical findings, associated with the severity of illness, increased significantly with the number of days to oseltamivir administration: abnormal respiratory sounds; hemoptysis; pulmonary infiltrative shadows, pneumothorax and pleurisy on chest radiographs; dyspnea; cyanosis; and need for intubation (Table 2). Conversely, SpO_2_ decreased with time to oseltamivir administration, as did the incidence of upper respiratory tract symptoms, including nasal obstruction, sore throat and conjunctivitis (Table 3). These findings suggest that the viral infection began in the upper respiratory tract and descended to the lower respiratory tract, and then the virus proliferated in the alveolar lung tissues in most pneumonia.

In this study, severity of respiratory features ranged from upper respiratory tract involvement without pneumonia to severe pneumonia. The proportions of patients with severe pneumonia and mild to moderate pneumonia increased from Groups 1 to 4. (Table 4). By contrast, the proportions of patients with upper respiratory tract involvement without pneumonia decreased from Groups 1 to 4. The association between severity of respiratory features and time to oseltamivir administration was found to be highly significant even after adjusting for socioeconomic levels, gender, hypertension, obesity, asthma and smoking (Table 5). Analysis of the probabilities of developing pneumonia indicated that the occurrence of any severity of pneumonia as well as that of severe pneumonia was associated with a longer interval from symptom onset to oseltamivir administration (Figure 1). The probability of occurrence of any severity of pneumonia was 50% at 3.4 days. The probability of severe pneumonia increased gradually and was approximately 10% at 3 days after symptom onset. These results indicate that early initiation of oseltamivir administration after the symptom onset is the significant factor for the reduction of severity of pneumonia. The probability of occurrence of severe pneumonia and any severity of pneumonia at 48 hours were <10% and <50%, respectively, and increased gradually in this study. The estimations suggest that oseltamivir administration, even >2 days following symptom onset, still had considerable potential for reducing the occurrence of pneumonia of any severity. This study also found that the shorter the delay in starting oseltamivir, the shorter the length of hospitalization (Figure 2). This finding is compatible with previous studies [14], [15].

Oseltamivir is a neuraminidase inhibitor that prevents cleavage of sialic acid on the surfaces of host cells, thus preventing new viral particles from being released by infected cells [10]. Viral neuraminidase is also known to play a role in the initial stages of airway epithelial cell infection [16] and to assist in hemagglutinin fusion [17]. Therefore, oseltamivir reduces viral cell entry as well as release. Seasonal influenza has been shown to replicate in humans for approximately 7 days post infection [18]. In pandemic H1N1 2009, it is likely that either the virus had an evasive phenotype [19] or the patient was predisposed to severe disease. This may lead to the assumption that patients who respond to oseltamivir at a later time after symptom onset still harbor actively replicating influenza virus [20]. This study might include patients who had difficulty in controlling influenza virus replication once it had taken hold within patients' bodies. The study also might constitute the population who has various host responses to the novel H1N1 influenza virus.

An important issue revealed in this study is the late access to medical care in Mexico. One of the main reasons for this may have been the cost consciousness of the patients; another may have been a lack of education. Such issues may have strongly influenced the severity of illness and need to be further investigated.

The present study has several limitations. First, the study was retrospective and there were no clear criteria for oseltamivir administration when patients presented. Second, there were also patients who were not administered oseltamivir and were excluded from this study. Since we only included patients administered oseltamivir, there is a potential bias. Most patients with influenza recovered without antiviral treatment. The study patients, who visited the INER, were likely to be representative of those patients with more severe disease. Third, some patients may have had secondary bacterial infection in addition to primary viral infection [3]. This study was also unable to evaluate bacterial infection or antibiotic treatments because of insufficient data.

Regardless, the findings of this study will be valuable across many fields of medicine, science, and public health. Clinically, the present results support the initiation of treatment as soon as a patient presents, regardless of whether 48 hours have passed since disease onset. In addition, the effect of oseltamivir on the occurrence and severity of pneumonia shows that inhibition of infection of new cells is linked to better clinical outcomes. Moreover, these findings will help planning for future pandemics and may result in an increase in the availability of oseltamivir treatment to individuals in the community who have not recovered significantly after 2 days when afflicted with influenza-like illness.

In conclusion, the present findings indicate that earlier initiation of oseltamivir administration after symptom onset significantly reduced the occurrence and severity of pneumonia and shortened the length of hospitalization in patients with pandemic H1N1 2009. In addition, even when administered >48 hours after symptom onset, oseltamivir showed considerable potential to reduce the occurrence and severity of pneumonia. The results from this retrospective study which indicated the effectiveness of earlier administration of antiviral agents at any time after symptom onset for reducing the occurrence of pneumonia should benefit patients affected by the next influenza pandemic. However, continued investigation and further prospective studies to more fully define the effectiveness of early antiviral treatment are required.
